# Novel Respiratory Syncytial Virus (RSV) Genotype ON1 Predominates in Germany during Winter Season 2012–13

**DOI:** 10.1371/journal.pone.0109191

**Published:** 2014-10-07

**Authors:** Julia Tabatabai, Christiane Prifert, Johannes Pfeil, Jürgen Grulich-Henn, Paul Schnitzler

**Affiliations:** 1 Department of Infectious Diseases, Virology, University of Heidelberg, Heidelberg, Germany; 2 London School of Hygiene and Tropical Medicine, London, United Kingdom; 3 Institute of Virology and Immunobiology, University of Würzburg, Würzburg, Germany; 4 Department of Pediatrics, University of Heidelberg, Heidelberg, Germany; 5 German Centre for Infectious Diseases (DZIF), Heidelberg, Germany; University of Iowa, United States of America

## Abstract

Respiratory syncytial virus (RSV) is the leading cause of hospitalization especially in young children with respiratory tract infections (RTI). Patterns of circulating RSV genotypes can provide a better understanding of the molecular epidemiology of RSV infection. We retrospectively analyzed the genetic diversity of RSV infection in hospitalized children with acute RTI admitted to University Hospital Heidelberg/Germany between October 2012 and April 2013. Nasopharyngeal aspirates (NPA) were routinely obtained in 240 children younger than 2 years of age who presented with clinical symptoms of upper or lower RTI. We analyzed NPAs via PCR and sequence analysis of the second variable region of the RSV G gene coding for the attachment glycoprotein. We obtained medical records reviewing routine clinical data. RSV was detected in 134/240 children. In RSV-positive patients the most common diagnosis was bronchitis/bronchiolitis (75.4%). The mean duration of hospitalization was longer in RSV-positive compared to RSV-negative patients (3.5 vs. 5.1 days; p<0.01). RSV-A was detected in 82.1%, RSV-B in 17.9% of all samples. Phylogenetic analysis of 112 isolates revealed that the majority of RSV-A strains (65%) belonged to the novel ON1 genotype containing a 72-nucleotide duplication. However, genotype ON1 was not associated with a more severe course of illness when taking basic clinical/laboratory parameters into account. Molecular characterization of RSV confirms the co-circulation of multiple genotypes of subtype RSV-A and RSV-B. The duplication in the G gene of genotype ON1 might have an effect on the rapid spread of this emerging RSV strain.

## Introduction

Respiratory syncytial virus (RSV) is the major pathogen of lower respiratory tract infections (RTI) in infants and young children. By the age of 2 years, virtually all children have been infected at least once with RSV [1]. Re-infections are common throughout life; in older children and adults infections are associated with milder disease indicating that RSV induces only partial immunity [2]. Strain variation is thought to contribute to its ability to cause frequent re-infections [3] enabling RSV to remain present at high levels in the population [4]. Viral strains are separated into two major groups based on its genetic and antigenic variability. Several lineages within the subtypes RSV-A and RSV-B co-circulate simultaneously in the population [5] and their relative proportions may differ between epidemics, although RSV-A viruses tend to predominate [6]. The main differences between RSV-A and RSV-B are found in the attachment (G) glycoprotein [7]. The G protein is a type II surface glycoprotein of about 300 amino acids in length, consisting of a cytoplasmic domain, a transmembrane domain and an ectodomain. The G protein is heavily glycosylated with N-linked and O-linked sugars. However, the amino acid sequence positions of potential glycosylation sites are poorly conserved [8].

This protein is able to accommodate drastic changes with the emergence of new variants. Diversity occurs mainly in the two hypervariable regions of the ectodomain which are separated by a highly conserved 13-amino acid (aa) length domain [9]. Sequencing of the second hypervariable region at the C-terminal end of the G gene has been widely used to further subdivide RSV-A and RSV-B into genotypes and facilitated differentiation between RSV isolates. To date, 11 RSV-A genotypes, GA1-GA7, SAA1, NA1-NA2, and ON1 [10]–[12], and 23 RSV-B genotypes, GB1-GB4, SAB1-SAB3, SAB4, URU1, URU2, BAI - BAXII, and THB [10], [13]–[21] have been described based on nucleotide sequence analysis.

RSV strains show an accumulation of translated amino acid changes over the years, suggesting antigenic drift-based immunity-mediated selection [22]. In 1999, a new RSV-B genotype BA emerged in Buenos Aires, Argentina, containing a 60-nucleotide (nt) duplication in the second hypervariable region of the G gene [23]. In the following ten years, the BA genotype spread worldwide and largely replaced previous described RSV-B genotypes [24]. During the 2010–11 winter season, a novel RSV-A genotype ON1 with a 72-nt duplication has been reported in Canada [14]. In line with the gradual spread of the BA genotype making it the dominant circulating RSV-B genotype today, the nucleotide duplication of the ON1 genotype might likewise result in a similar selection advantage [25]. There is an increasing number of reports from across the world describing this novel genotype and the following seasons will show its impact on the evolution of RSV-A [6].

In Germany, there is only limited information regarding the molecular epidemiology of RSV, the emergence of novel viral strains and their impact on the course of RSV infection. In the present study, we evaluated hospitalized children below the age of 2 years presenting with acute RTI in the Pediatric Department in Heidelberg, Germany during the winter season 2012–13. We investigated the genetic diversity and patterns of the co-circulating genotypes of Heidelberg RSV-A and RSV-B strains in comparison with other RSV strains circulating worldwide. Furthermore we explored a possible association between individual RSV genotypes and the course of RSV infection by retrospectively analyzing basic clinical and laboratory data.

## Materials and Methods

### Patients and clinical samples

We retrospectively analyzed children under the age of 2 years admitted to the Pediatric Department at the Heidelberg University Hospital between October 2012 and April 2013 with clinical symptoms of upper or lower respiratory tract infection (RTI) as part of their admission diagnosis or as concomitant symptom. Prior to the transfer to the inpatient unit, nasopharyngeal aspirates (NPA) are obtained and these children are routinely screened for RSV infection using a rapid antigen test in order to inform for isolation strategies. All obtained NPAs (242 samples from 240 children) were collected and stored frozen for further molecular analysis by RSV PCR and phylogenetic analysis. Medical records were reviewed from all children to obtain routine clinical and laboratory data. Patient records were anonymized and de-identified prior to analysis. The Ethical Committee of the University of Heidelberg has approved this study (S-166/2014).

### PCR and sequencing

For PCR analysis, RNA was extracted from NPAs using the QIAamp viral RNA mini kit (Qiagen, Hilden, Germany) according to the manufacturer's protocol. Amplification and detection of viral RNA was performed with the RealStar RSV real-time PCR kit (altona Diagnostics, Hamburg, Germany) on a LightCycler 480 instrument II (Roche, Mannheim, Germany). This assay differentiated RSV into subtypes A and B. For sequencing and identification of RSV genotypes, extracted RNA was initially reverse transcribed and cDNA was synthesized using random hexamer primers. Subsequently, PCR targeting the second hypervariable region of the G gene was performed using primer pairs as previously described by Peret et al. [11]. PCR products were sequenced with the same primer pairs previously used for amplification. Overlapping sequences were assembled and edited using the SEQMAN II software of the Lasergene package (DNAstar, Madsion, WI). Nucleotide sequences of the second hypervariable region of the G gene retrieved in this study were deposited in GenBank under accession numbers [KJ710364-KJ710420].

### Phylogenetic and deduced amino acid sequence analysis

Multiple sequence alignments and phylogenetic analysis of the second hypervariable region of the G gene were conducted using the Clustal W 1.6 method of MEGA software version 6 [26]. Phylogenetic trees were generated using the maximum-likelihood method and bootstrap values with 1,000 replicates were calculated to evaluate confidence estimates. Reference strains representing known genotypes were retrieved from GenBank (http://www.ncbi.lm.nih.gov) and included in the tree. Pairwise nucleotide distances were calculated to compare the differences within and between genotypes of subgroup RSV-A and RSV-B using MEGA software version 6. Positive selected sites were estimated by use of the Datamonkey Web server (http://www.datamonkey.org) identifying the rates of non-synonymous and synonymous substitutions [27].

Deduced amino acid sequences were translated with the standard genetic code using MEGA software version 6. Alignments of the second hypervariable region of the G protein of Heidelberg RSV-A and RSV-B strains were compared to references strains from GenBank to identify amino acid substitutions. Putative N-glycosylation sites were predicted if the encoded amino acid sequence was Asn-Xaa-Thr/Ser, where Xaa was not a proline and accepted if the glycosylation potential was ≥0.5 in NetNGlyc 1.0 server [28]. O-glycosylation was determined using the NetOGlyc 3.1 server and sites were predicted using a G-score ≥0.5 [29].

### Statistical analysis of epidemiological factors

To describe the temporal distribution of admitted RSV cases, we aggregated RSV results as obtained by PCR by calendar month and week. Demographic and clinical data in our study population was summarized. Group comparisons were performed using χ2 or Fisher's exact test for categorical variables and by Student's t-test or analysis of variance (ANOVA) for continuous variables, as appropriate. P-values <0.05 were considered statistically significant. Stata/IC13.0 (StataCorp. LP, College Station, TX, USA) was used for all statistical analysis.

## Results

### Detection of RSV

**Figure 1:**
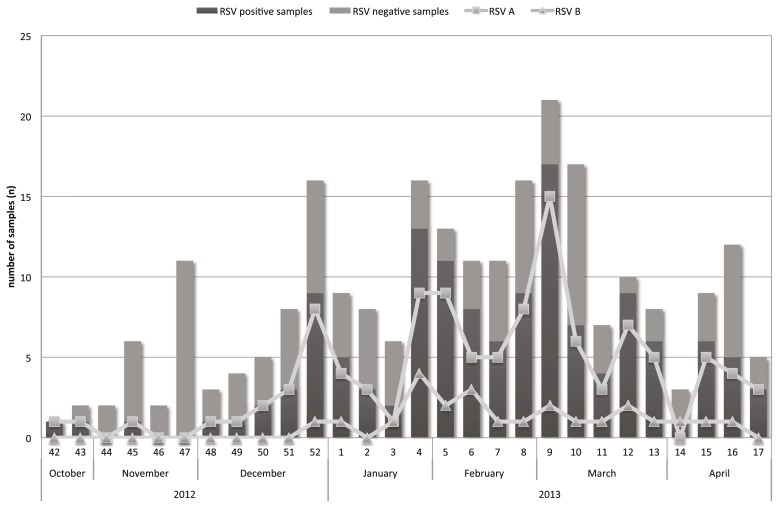
Weekly/monthly distribution of subgroup RSV A/RSV B in children ≤2 years with acute RTI in Heidelberg/Germany, winter season 2012/13.

Between October 2012 and April 2013, a total of 242 samples from hospitalized infants and children were analyzed for RSV infection by PCR resulting in 134 (55.4%) RSV-positive samples. Among these RSV-positive samples, 110 (82.1%) were sub-grouped as RSV-A and 24 (17.9%) as RSV-B, respectively. No co-infection for RSV-A and RSV-B was detected. Two children presented twice and were tested RSV-positive at their first admission and RSV-negative at the consecutive admission few weeks later. The distribution of RSV-A and RSV-B per calendar week and month is shown in Fig. 1.

### Sequence alignments and phylogenetic analysis

**Figure 2:**
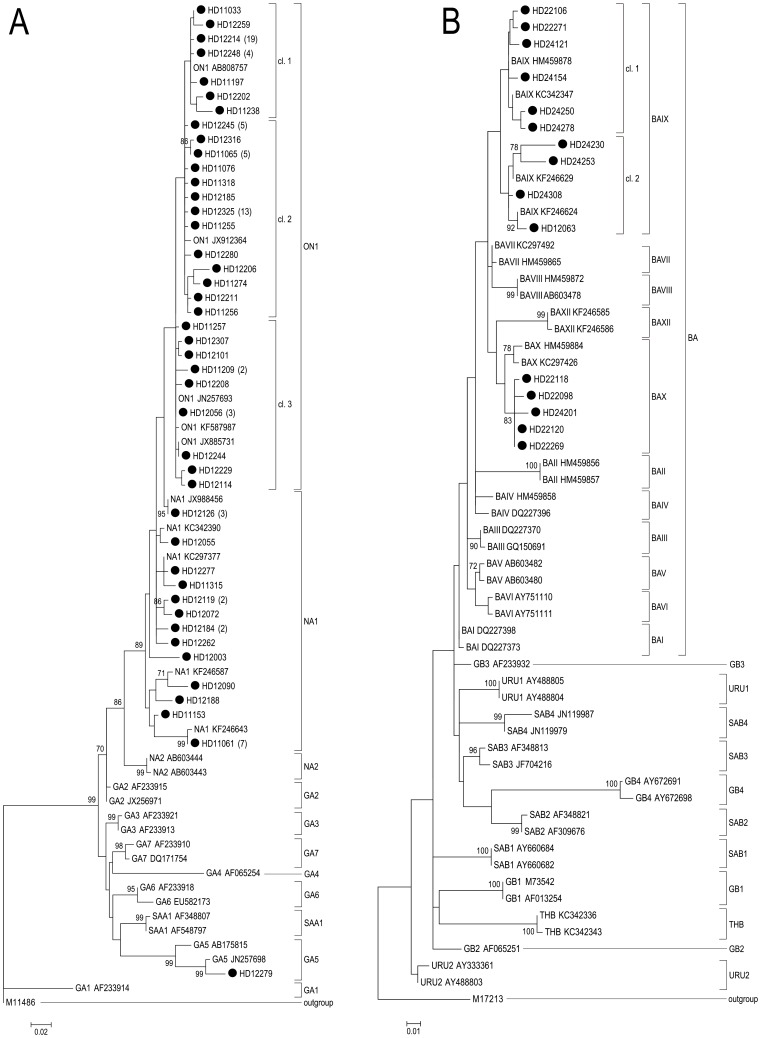
Phylogenetic tree of RSV A/RSV B strains and reference sequences of identified genotypes. Phylogenetic trees for RSV A (A) and RSV B (B) strains were constructed with maximum-likelihood method with 1,000 bootstrap replicates using MEGA 6 software. RSV strains from Heidelberg/Germany are indicated by “•HD” followed by their strain identification number. Number of identical strains is indicated in brackets after the strain identifier. Reference strains representing known genotypes were retrieved from GenBank and included in the tree (labels include accession number). The genotype assignment is shown on the right by brackets. Prototype strains (M11486 for subgroup A and M17213 for subgroup B) were used as an outgroup. Bootstrap values greater than 70% are indicated at the branch nodes. The scale bar represents the number of nucleotide substitutions per site. cl.  =  cluster.

Sequences of the second hypervariable region of the G gene from 97 (72.4%) RSV-A and 15 (11.2%) RSV-B samples were successfully obtained and aligned with representative GenBank sequences of previously published genotypes. Due to a low viral load some RSV-positive samples (n = 22; 16.4%) could not be sequenced. The phylogenetic trees of RSV-A and RSV-B sequences are shown in Figure 2. Heidelberg RSV_A and RSV-B strains clustered into three genotypes for RSV-A and two genotypes for RSV-B, respectively.

The majority of RSV-A strains (n = 73, 75.3%) clustered with strains that were previously assigned to the novel ON1 genotype with a 72-nt duplication, followed by strains clustering with genotype NA1 (n = 23, 31.5%) and one strain clustering with GA5 (Fig. 2A). Sequence homology between Heidelberg sequences and the ON1 reference strain [JN257693] was ≥96.9% at the nucleotide level and ≥94.1% at the amino acid level. The intragenotypic p-distance was 1.9% for ON1 and 6.2% for NA1 for Heidelberg sequences. The intergenotypic p-distance for ON1 and NA1 was not comparable because of the 72-nt duplication.

All RSV-B strains (n = 15) clustered with strains that were previously assigned to the BA genotype with a 60-nt duplication. In addition, BA strains could be further differentiated into the previously designated genotypes BAIX (n = 10, 66.7%) and BAX (n = 5, 33.3%) (Fig. 2B). Sequence homology between Heidelberg sequences and the BA reference strain (AY333364) was 94.3%–96.6% at the nucleotide level and 87%–95% at the amino acid level. The intergenotypic p-distance for BAIX and BAX was 4.7% for Heidelberg sequences, with an intragenotypic p-distance of 3% for BAIX and 0.8% for BAX.

### Deduced amino acid sequence analysis

**Figure 3:**
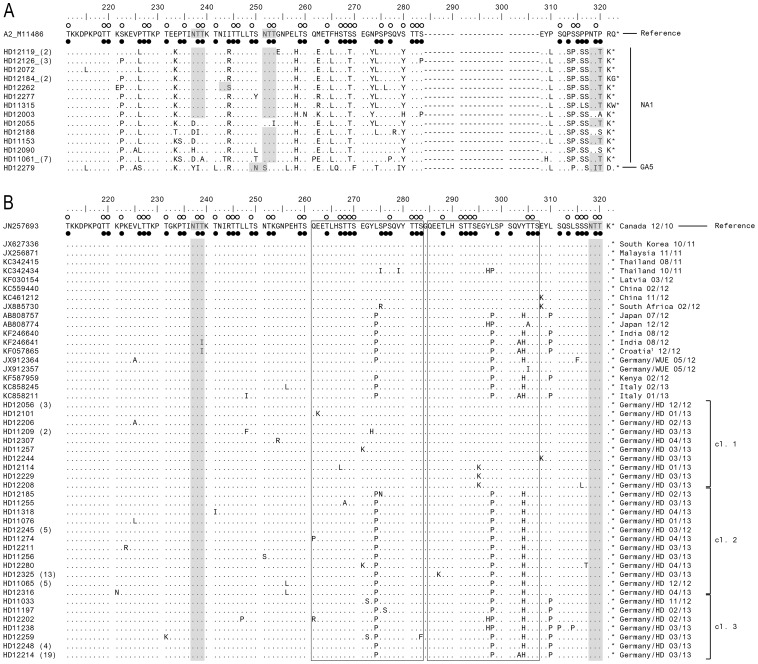
Alignment of deduced amino acid sequences of RSV-A strains. A) Alignment of RSV-A genotype NA1 and GA5 are shown relative to the sequence of prototype strain A2 (GenBank accession number M11486). Alignment of sequences was performed using the Clustal W 1.6 method via MEGA 6 software. The amino acid positions correspond to positions 210 to 298 of the G protein of strain A2. Identical residues are indicated by dots, asterisks indicate the position of stop codons. Number of identical strains is indicated in brackets after the strain identifier in the left column. Gray shading highlights predicted N-glycosylation sites. Unfilled circles indicate predicted O-glycosylation sites of the prototype strain A2; potential O-glycosylation sites of Heidelberg strains are indicated by black dots. The genotype assignment is shown on the right by brackets. B) Alignments are shown relative to the sequence of ON1 strain first described in Canada (GenBank accession number JN257693). Alignment of sequences was performed using the Clustal W 1.6 method via MEGA 6 software. The amino acid positions correspond to positions 210 to 298 of the G protein of the prototype strain A2. Identical residues are indicated by dots, asterisks indicate the position of stop codons. Boxes frame the 23 amino acid duplicated region of the 24 amino acid insertion. Gray shading highlights predicted N-glycosylation sites. Unfilled circles indicate predicted O-glycosylation sites of the Canadian reference ON1 strain; potential O-glycosylation sites of Heidelberg strains are indicated by black dots. On the right hand site, GenBank and Heidelberg strains are labeled with the country and time of occurrence (month/year). ^1^ Sequences were published in GenBank only. HD =  Heidelberg; WUE =  Wuerzburg, cl. =  cluster.

We aligned and compared Heidelberg RSV-A genotype NA1 and GA5 strains with the A2 reference strain (M11486) (Fig. 3A). The majority of NA1 strains had three predicted N-glycosylation sites at amino acid (aa) positions 237, 251 and 294. However, HD12262 had an additional predicted N-glycosylation site at aa position 242 and HD12188 at aa position 273. A group of 11 isolates lost the N-glycosylation site at aa positions 237 due to a N237D/H substitution. Strain HD12055 lost all N-glycosylation site but the aa position 294. The N-glycosylation site at aa position 250 is characteristic for the GA5 genotype as found in one of the Heidelberg isolates. O-glycosylation patterns varied between Heidelberg isolates with 32±3 sites potentially O-glycosylated (G-score ≥0.5) in NA1 isolates and 23 sites in the one GA5 isolate.

As a consequence of the insertion in the G gene, ON1 genotype strains translate into a polypeptide with a length of 321 amino acids and are thereby lengthened by 24 aa when compared to the A2 reference strain (Fig. 3B). The insertion contains a 23 aa duplicated region. The comparison of Heidelberg ON1 strains with ON1 strains from other countries with reference to the original strain from Canada (JN257693) revealed that Heidelberg isolates can be divided into three sub-clusters: The first sub-cluster (n = 12) comprises isolates closely related to the primary Canadian strain (JN257693) with few mixed substitutions. This sub-cluster also includes one strain with an E308K substitution as previously described in South African strains [JX885730]. The second sub-cluster (n = 28) contained three characteristic substitutions, L274P, L298P and Y304H as previously described in strains from Wuerzburg, Germany [JX912364, JX12364], as well as in strains from Italy and Japan [KC858245, KC587959; AB808774, AB808757]. Furthermore, on group of 13 identical isolates showed a E287K substitution which was unique for Heidelberg strains. The third sub-cluster consists of 28 isolates with a L310P substitution in addition to the three substitutions found in the second sub-cluster and aligned with isolates from Japan, India and Kenya [AB808774, AB808757; KF246641, KF246640; KF587959]. A group of 19 identical strains additionally showed a V303A substitution, which was also seen in strains from Italy, Croatia and India [KC858245, KC587959; KF057865; KF246641, KF246640]. However, positive selection analysis of ON1 strains revealed no positive selected site.

All Heidelberg ON1 strains had two predicted N-glycosylation sites at aa positions 237 and 318 and lost the N-glycosylation sites at aa position 251 due to a T251K substitution. ON1 strains showed different patterns of O-glycosylation sites with 31 to 44 predicted sites and showed some new sites when compared to the Ontario reference strain. The 23-aa duplicated region contained a maximum of 10 glycosylation sites as observed in 17 ON1 isolates from Heidelberg.

**Figure 4:**
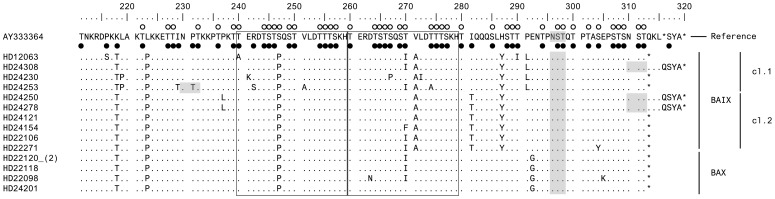
Alignment of deduced amino acid sequences of the second variable region of the G protein of RSV-B strains isolated in Heidelberg/Germany during 2012–2013 winter season. Alignments are shown relative to the sequence of a prototype BA strain (GenBank accession number AY333364). Alignment of sequences was performed using the Clustal W 1.6 method via MEGA 6 software. The amino acid positions correspond to positions 210 to 315 of the G protein of the BA strain. Identical residues are indicated by dots, asterisks indicate the position of stop codons. Number of identical strains is indicated in brackets after the strain identifier in the left column. Boxes frame the 20 amino acid duplication. Gray shading highlights predicted N-glycosylation sites. Open circles indicate predicted O-glycosylation sites of the prototype BA strain; potential O-glycosylation sites of Heidelberg strains are indicated by black dots. Genotypes are shown on the right by brackets.

Heidelberg RSV-B genotype BA strains were compared to BA reference strain from Buenos Aires [AF33364] (Fig. 4). Stop codons were either at aa position 320 for HD24308, HD24250 and HD24278 or at aa position 313 for the remaining BA strains. All BA strains had a K218P, L223P and S247P substitution. The BAIX genotype had a H287Y and a V271A substitution. Furthermore some BAIX strains (sub-cluster 1) had a P291L substitution whereas other BAIX strains (sub-cluster 2) had an I281T substitution. The BAX strains had a P291G substitution.

All Heidelberg RSV B strains had a predicted N-glycosylation site at aa positions 296. However the N-glycosylation site at aa position 310 only fulfilled the typical amino acid patterns but the glycosylation potential calculated by NetOGlyc 3.1 server was below 0.5 in isolates with a stop codon at aa position 313. HD24253 had an additional potential N-glycosylation site at aa position 230. O-glycosylation of BA strains varied between 40 and 47 predicted sites and showed an additional predicted site at aa position 317 in longer strains with a stop codon at aa position 320.

### Basic and clinical characteristics of the study cohort

The mean age of the all screened children was 7.9 months and ranged in line with the inclusion criteria between 11 days and 23.8 months. RSV-positive patients were significantly younger compared to RSV-negative patients (t-test; p<0.001). The age group distribution showed that 62.7% RSV-positive children were below 6 months of age. All children had at least a concomitant acute RTI at time of admission; however, in some patients the main clinical diagnosis was non-respiratory (total 12.4%, RSV positive 4.5%). In RSV-positive patients the most common diagnosis was bronchitis/bronchiolitis (75.4%). The mean duration of hospitalization was longer in RSV-positive patients (3.5 vs. 5.1 days; p<0.01).

We performed a group comparison of the main three genotypes (RSV-A: ON1 and NA1; RSV-B: BA) using basic and clinical characteristics of RSV-positive infants and children (Table 1). There were no risk factors for one of the three groups of genotypes identified when looking at the demographic characteristics (age, gender, weight). Furthermore, we could not identify any association between a specific genotype and a more severe course of illness when taking the retrospectively available clinical and laboratory parameters into account.

**Table 1:** Basic and clinical characteristics of RSV positive children by genotype.

		RSV A*		RSV B	
	RSV positive	ON1	NA1	BA	p-value
	n = 134	n = 73	n = 23	n = 15	
**Demographic characteristics**					
Age in months, mean ±SD	6.5±6.2	6.4±6.2	5.7±5.6	4.8±5.1	0.63
Age group, n (%)					
0–6 months	84 (62.7)	48 (65.8)	15 (65.2)	10 (66.7)	0.18
>6–12 months	20 (14.9)	7 (9.6)	6 (26.1)	3 (20.0)	
>12–18 months	20 (14.9)	13(17.8)	0 (0.0)	2 (13.3)	
>18–24 months	10 (7.5)	15 (6.9)	2 (8.7)	0 (0.0)	
Gender, n (%)					
Male	83 (61.9)	44 (60.3)	18 (78.3)	9 (60.0)	0.28
Female	51 (38.1)	29 (39.7)	5 (21.7)	6 (40.0)	
Weight in kg, mean ±SD	6.2±2.3	5.9±2.1	7.0±2.7	5.8±2.3	0.11
**Leading clinical diagnosis, n (%)**					
Non-respiratory	6 (4.5)	4 (5.5)	0 (0.0)	1 (6.7)	0.49
Respiratory	128 (95.5)	69 (94.5)	23 (100.0)	14 (93.3)	
Upper RTI	4 (3.0)	1 (1.4)	1 (4.4)	0 (0.0)	0.61
Bronchitis/Bronchiolitis	101 (75.4)	54 (74.0)	20 (87.0)	12 (80.0)	
Pneumonia	23 (17.2)	14 (19.2)	2 (8.7)	2 (6.7)	
**Course of disease**					
Symptoms prior to hospitalization in days, mean ±SD	4.2±3.6	3.9±3.1	3.9±2.9	3.8±5.0	0.98
Hospital stay in days, mean ±SD#	5.3±3.9	5.3±3.8	5.5±2.8	4.5±2.1	0.68
Need for intensive care, n (%)	6 (4.5)	4 (5.5)	0 (0.0)	1 (6.7)	0.49
**Laboratory parameters on admission**					
Hemoglobin in g/dl, mean ±SD	11.7±1.7	11.4±1.5	11.9±1.9	11.7±2.0	0.45
Leucocytes/nl, mean ±SD	11.5±4.7	11.0±4.5	10.1±3.6	10.4±3.8	0.64
Thrombocytes/nl, mean ±SD	434.2±120.5	435.8±130.9	391.5±84.6	467.9±117.4	0.17
C-reactive protein in mg/L, mean ±SD	22.5±37.0	20.1±26.7	22.4±24.5	17.5±17.7	0.88
pCO2 in mmHg, mean ±SD	41.5±8.8	41.6±9.4	42.1±8.4	45.6±8.9	0.44

*RSV A genotype GA5 was not included in this table as this genotype was only present in one patient. In total, 112 of 134 RSV positive patients could be sequenced and a genotype could be determined.

# Hospital stay was only calculated for patients who stayed at least 24 hours in hospital.

SD =  standard deviation; RTI = respiratory tract infection, RSV =  Respiratory Syncytial Virus.

## Discussion

RSV accounts for a significant burden of acute respiratory tract infections particularly in infants and young children in need for hospital care [30]. Patterns of circulating RSV genotypes can provide a better understanding of the molecular epidemiology of RSV infection. In our study, we analyzed the genetic diversity and patterns of co-circulating genotypes of both, subtypes RSV-A and RSV-B, during the winter season 2012–13 in Heidelberg/Germany. RSV was detected in 134 out of 242 samples of which 110 (82.1%) were sub-grouped as RSV-A and 24 (17.9%) as RSV-B, respectively. Phylogenetic analysis revealed that the majority of RSV-A strains (n = 73, 75.3%) clustered with strains of the novel ON1 genotype with a 72-nt duplication first described by Eshaghi et al. in Ontario, Canada in 2010 [14]. In Germany, circulation of this genotype was reported for the first time in Wuerzburg in winter 2011–12 [31]. In line with another study in Europe, this study reports ON1 as the predominant genotype during the RSV epidemic season 2012–13, suggesting a rapid spread of this emerging RSV strain [32], [33].

Most RSV cases were detected between December 2012 and April 2013, which is in line with the previously described seasonality of RSV infection in Germany [30]. However, the core season with more than half of all RSV-positive cases was from end-January to mid-March, which can be considered a late pattern of a RSV epidemic season in Germany [22]. Within our study population RSV was detectable in 55.4% of hospitalized children below the age of 2 years emphasizing the need for RSV screening on admission to assure proper management and to prevent nosocomial infections [34].

In our cohort, age group analysis revealed that infants below 6 months of age had the highest infection rates, as expected. Primary RSV infection commonly occurs within the first year of life [13] and the risk of RSV infection decreases with increasing age [22]. The majority of RSV-positive children presented with bronchitis/bronchiolitis followed by pneumonia and the duration of hospitalization was significantly longer compared to RSV-negative patients. However, the median duration of hospital stay of 5 days in RSV-positive patients in this study was shorter compared to previous findings of 7 days [30].

Molecular analysis of RSV-positive samples demonstrated that RSV-A was the predominant subtype which is in line with previous findings of multiple-season studies from Europe and other geographical areas [21], [22]. Phylogenetic analysis revealed that the majority of RSV-A strains (n = 73, 75.3%) clustered with strains of the novel ON1 genotype with a 72-nt duplication. Phylogenetic analysis of Tsukagoshi et al. estimated that genotype ON1 evolved from genotype NA1 [35]. Recent estimates place the time of the ON1 emergence around 2008/09 [36], [37].

Over the past three epidemic seasons (2010–2013) the prevalence of ON1 strains among all circulating strains varied between the different reports, but there seems to be a trend towards ON1 as the predominating RSV-A strain. In its first description in Ontario/Canada, the genotype ON1 accounted for 10% of RSV-positive samples in the season 2010-11 [14]. In the same season in Thailand, the majority of RSV isolates belonged to NA1, only few isolates belonged to ON1 [20]. One season later in 2011–12, RSV-A genotype ON1 was reported in different studies from Asia, Africa and Europe suggesting a worldwide emergence of the novel RSV-A strain. However, ON1 was only sporadically detected during that time and some countries like Pakistan did not report ON1 among circulating genotypes in 2011–12 [34]. A study from Heidelberg/Germany evaluating the genetic diversity of RSV in an outbreak in a haematology unit in 2012 also did not describe any ON1 strains [38]. In a study from Bejing, China, only one sample from February 2012 out of about 250 sequenced RSV-positive samples between 2007 and 2012 was characterized as ON1 genotype [39]. One report from Wuerzburg/Germany, assigned 10% of the identified strains to genotype ON1 in 2011–12 [31]. In the past months an increasing number of reports about circulating genotypes in the season 2012–13 were published. In line with the findings in this study, reports from Cyprus, Italy, Kenya and South Korea described ON1 as the predominating genotype in the epidemic season 2012–13 [32], [33], [36], [37]. Our study therefore describes a further cohort in Europe with ON1 as the predominating genotype in 2012–13 suggesting a rapid emergence of this novel strain. Further surveillance of circulating genotypes will be needed to observe the future global distribution of the ON1 strain and its trend to diversify.

The comparison of Heidelberg ON1 strains with ON1 strains from other countries revealed that Heidelberg isolates could be divided into three sub-clusters with characteristic substitutions as previously described in different countries and continents. Similar to the subdivision of RSV-B genotype BA into several sub-genotypes (BA-I – BAXII), this could be a first trend to a diversification of the ON1 genotype. However, none of the sub-clusters had bootstrap values ≥70%. Furthermore, positive selection analysis did not reveal any positively selective sites among ON1 isolates. This is also reflected by two groups of identical isolates (n = 13 and n = 18) of the ON1 genotype, suggesting the absence of selective pressure among the newly emerging strains.

Although the majority of strains was subtyped as RSV-A, 13.4% of all Heidelberg trains were subtyped as RSV-B. All RSV-B strains clustered with strains of the BA genotype with a 60-nt duplication, first described by Trento et al. in Buenos Aires, Argentina in 1999 [23], further differentiating into the genotypes BAIX (n = 10, 66.7%) and BAX (n = 5, 33.3%). Genotype BAIX separated into two sub-clusters: one including the BAIX reference sequence from Japan designated by Dapat et al. [18] who first described this genotype in 2007, and the second including the BAIX reference sequence from India described by Choudhary et al. in 2010 [40]. However, none of the two sub-clusters had bootstrap values ≥70%.

Similar to genotype BA, the nucleotide duplication of the ON1 genotype seems to result in a selection advantage compared to other RSV-A genotypes. Interestingly, despite the emergence of the ON1 virus there is conflicting data concerning the virulence in terms of disease severity [31]–[33]. In our cohort, retrospective analysis of basic clinical and laboratory parameters such as the duration of hospital stay, the need for intensive care as well as pCO_2_ levels on admission did not reveal any association between a specific genotype and disease severity. Further surveillance of circulating RSV genotypes and corresponding clinical data is needed to understand the evolution, transmission and pathogenicity of genotype ON1 RSV infections.

Our study is subject to several limitations: We report the genetic diversity of RSV during one season in winter 2012–13 and a comparison of proportions of circulating genotypes is therefore restricted to other reports from Germany as well as worldwide. Our analysis included hospitalized children with community acquired RSV infection and therefore cannot draw conclusions for the overall population of community acquired RSV infections. However, the cohort of hospitalized children is of particular clinical relevance. Due to the retrospective study design, the evaluation of the association between pathogenicity of RSV infection and genotypes was limited to the available data. Furthermore, we did not include an analysis of further co-infections, which might also have an effect on disease severity in the evaluated study cohort. Further surveillance of the molecular epidemiology for several seasons in combination with prospectively complied clinical data is needed to directly compare the emergence of new variants and their transmissibility and virulence.

In summary, molecular characterization of RSV in Heidelberg, Germany during winter season 2012–13 confirmed the co-circulation of multiple genotypes of subtype RSV-A and RSV-B and the predominance of the novel genotype ON1. In line with the emergence of the BA genotype, it can be hypothesized that genotype ON1 could spread in a similar way and several branches might subdivide into further sub-genotypes. However, we could not find any association between disease severity and this newly emerging RSV-A genotype ON1. Continuing and long-term molecular epidemiological surveys for early detection of circulating and newly emerging genotypes in combination with clinical data are necessary to gain a better understanding of underlying genetic and antigenic mechanisms of RSV infection.
